# Classical Nucleation Theory and Tolman Equation in Cluster Thermodynamics: How Small Can They Truly Apply?

**DOI:** 10.1021/acs.jpca.5c02843

**Published:** 2025-06-27

**Authors:** Bin Chen

**Affiliations:** Department of Chemistry, 5779 Louisiana State University, Baton Rouge, Louisiana 70803-1804, United States

## Abstract

Classical nucleation theory and the Tolman equation are two fundamental theories in cluster thermodynamics. Despite their long-standing existence, the applicability of these theories remains questionable. Direct experimental validation is challenging due to the small size of the clusters involved. While theoretical approaches are often used as alternatives, the findings are frequently controversial. In this work, free energy calculations were performed across an unprecedentedly large size range using sophisticated techniques, including aggregation-volume-bias Monte Carlo, for two systems: Lennard-Jones and TIP4*P*/2005 water. The availability of bulk-phase properties for an infinitely large system (i.e., γ^∞^) facilitates a direct comparison to these two theories. The simulation results provide strong support for the applicability of these theories to large clusters, down to those containing a few hundred particles. However, these theories break down for small clusters.

## Graphical abstract

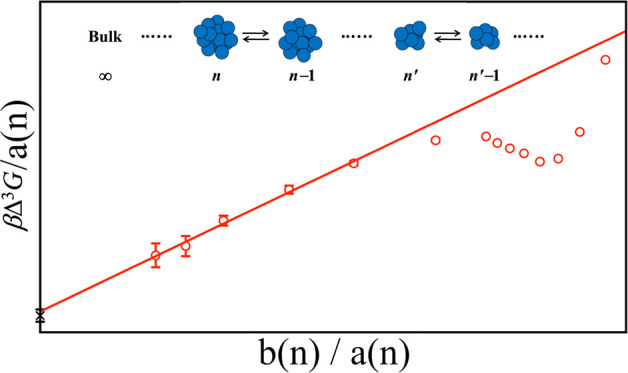

## 1 Introduction

Cluster thermodynamics studies the behavior of clusters of varying sizes and is crucial for understanding the nucleation process—the formation of embryos of a new phase from a metastable supersaturated mother phase—a phenomenon that plays a critical role in many natural and industrial processes. For many systems, classical nucleation theory (CNT), developed nearly a century ago by Volmer and Weber,^1^ Becker and Doering,^2^ and Zeldovich,^3^ has remained the dominant framework for describing the thermodynamics of cluster formation. By utilizing bulk-phase thermodynamic properties such as chemical potential (*μ*), surface tension (*γ*), and bulk density (*ρ*), CNT expresses the free energy of cluster formation (with *n* particles and a radius *R*) as the sum of a favorable bulk term and an unfavorable surface term, as follows

1 $$\Delta G\left(n\right)=n\Delta \mu +4\pi R^{2}\gamma =n\Delta \mu +n^{2/3}{\left(\frac{36\pi }{\rho^{2}}\right)}^{1/3}\gamma$$

In the above equation, the surface tension, *γ*, is taken to be the value of an infinitely planar surface *γ* ^∞^, and is assumed to remain constant regardless of the cluster size. However, this assumption contradicts Tolman’s seminal work,^4^ which demonstrated that the surface tension varies with the cluster size. Specifically, Tolman proposed that

2 $$\gamma =\gamma^{\infty }\left(1-\frac{2\delta }{R}\right)$$

where *δ* refers to the Tolman length. Thus, the assumption of size-independent surface tension (*γ*) in CNT has been identified as problematic, as discussed in numerous studies.^5–17^

While direct experimental validation of the Tolman equation remains challenging, theoretical approaches, such as classical density functional theory (c-DFT) and computer simulations, are commonly used as alternatives. For instance, c-DFT studies predict a negative Tolman length for both Lennard-Jones droplets and liquid water.^18–22^ In contrast, simulations and other theoretical approaches have yielded conflicting results, with both positive and negative Tolman lengths depending on the method used.^11,12,23–29^ For the TIP4*P*/2005 water model,^30^ the Tolman length was found to be negative using the mitosis method,^11,12^ while a positive Tolman length was observed with the test area method.^25^ The Tolman length was also shown to be positive by a simulation study that employed both thermodynamic and mechanical approaches to pressure calculation.^26^ Furthermore, some studies suggest a breakdown of the first-order Tolman equation,^31^ while others argue that surface tension should be independent of curvature, leading to a Tolman length of zero.^32^

In this work, the aggregation-volume-bias Monte Carlo method,^33,34^ developed for efficient nucleation simulations,^35–38^ was used to calculate the nucleation free energies of clusters across an unprecedentedly large range of sizes for two systems: Lennard-Jones and TIP4*P*/2005 water.^30^ These two systems are selected due to the availability of bulk-phase properties from an infinitely large system (i.e., *γ* ^∞^), which allows for a more direct validation of the two fundamental theories discussed above: classical nucleation theory and the Tolman equation.

## 2 Methods

This simulation study was made possible by combining aggregation-volume-bias Monte Carlo (AVBMC)^33,34^ with preferential selection of the interfacial region for particle swap moves^39^ and umbrella sampling.^40^ All simulations were performed using the grand canonical ensemble, where the cluster is physically isolated but thermodynamically coupled to a chemical potential bath (or an ideal gas phase at a specified density).^35^ To facilitate equilibration of the chemical potential, particle swap moves—enhanced by AVBMC with preferential selection of the interfacial region—were employed. Specifically, a target particle is preferentially selected near the cluster’s interfacial region based on an energy-based criterion, and a local volume is defined as a sphere with a radius of 1.5 σ for Lennard-Jones and 5 Å for water centered around this target particle. This local volume is also part of the cluster criterion, that is, two particles were considered part of the same cluster if one lay within the insertion volume of the other. In insertion moves, a new particle is transferred into this local volume to ensure that each successful insertion move leads to a growth of the cluster size by one. To improve the acceptance rate, a multiple-insertion strategy is used, where ten trial insertions are performed, and a Rosenbluth selection scheme^41^ frequently used in configuration-bias Monte Carlo^42–47^ biases the process toward the most favorable configuration, with the bias corrected using the Rosenbluth weight. For deletion moves, a particle (excluding the target particle) is selected from those inside the local volume and removed from the cluster (or added to the gas phase). To ensure reversibility, the Rosenbluth weight is calculated using this original old configuration and nine other randomly generated configuration within this local volume. Since particles with higher interaction energies are more likely to be removed, an energy-based selection scheme is used to choose both the target and the candidate particle for removal (see ref 39). In addition, umbrella sampling^40^ is used, where a biasing potential ensures that clusters of all sizes of interest are evenly sampled in the simulation. Translational moves (and rotational moves for TIP4*P*/2005 water) are also used to sample the system, with the moves equally divided among all types.

For comprehensive coverage of the free energy landscape, calculations are performed over a broad range of cluster sizes, specifically for clusters containing 20 ± 2, 40 ± 2, 80 ± 2, 200 ± 2, 400 ± 2, 800 ± 2, 2000 ± 2, 4000 ± 2, and 8000 ± 2 particles. This approach aims to interpolate and extrapolate the information using a finite set of clusters which is sufficient for validating the two theories mentioned above. All interactions are included, and each cluster is sampled at least 10^10^ times. The standard deviation was calculated by dividing the total simulation length into five blocks. The simulations were performed at *T* = 0.7 for Lennard-Jones and 300 K for TIP4*P*/2005 water, chosen based on the availability of *γ* ^∞^.

The initial configurations were either taken from previous simulations or generated by gradually growing small clusters under supersaturated conditions. Umbrella sampling began with the smallest cluster size (20 ± 2 molecules) for faster convergence, and the derived nucleation free energies were extrapolated to initialize biasing potentials for larger clusters. These potentials were iteratively refined until all cluster sizes were sampled uniformly (≤1% frequency variation). Production runs used 20–80 independent simulations with unique initial configurations and random seeds to enhance sampling efficiency. For Lennard-Jones, the results were reported in reduced units unless explicitly specified.

## 3 Results and Discussion

Figure 1 shows the ΔΔ*G* (or Δ^2^ *G*) results, specifically, Δ*G*(*n* + 2) – Δ*G*(*n* – 2), introduced in ref 48, plotted as a function of (*n* + 2)^2/3^ – (*n* – 2)^2/3^ obtained at *T* = 0.7 and *ρ*_v_ = 2.5 × 10^–3^ for Lennard-Jones, or at *T* = 300 K and *ρ*_v_ = 1 × 10^–6^ molecule/Å^3^ for TIP4*P*/2005 water. This plot has often been used to examine the applicability of CNT to cluster free energy predictions.^37,38,49–54^ From CNT or eq 1

$$\Delta^{2}G=\Delta G\left(n+2\right)-\Delta G\left(n-2\right)=4\Delta \mu +\left[{\left(n+2\right)}^{2/3}-{\left(n-2\right)}^{2/3}\right]{\left(\frac{36\pi }{\rho^{2}}\right)}^{1/3}\gamma$$ 3

**Figure 1:**
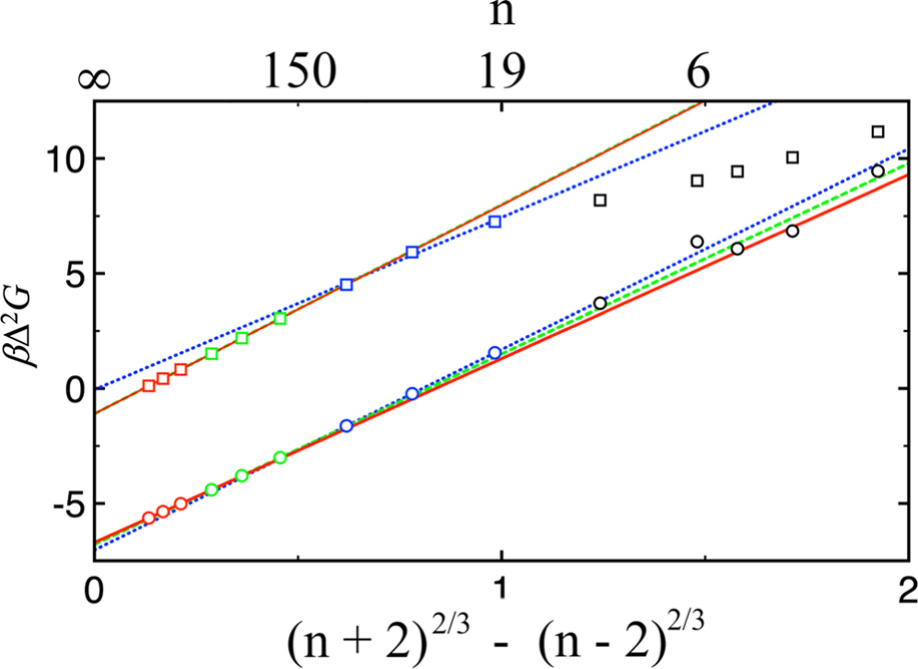
Δ^2^ *G* (=Δ*G*(*n* + 2) – Δ*G*(*n* – 2)) in units of *k*_B_ *T* as a function of (*n* + 2)^2/3^ – (*n* – 2)^2/3^ obtained for Lennard-Jones (squares) and TIP4*P*/2005 water (circles). Linear fits performed over the three cluster size ranges are shown as blue dotted lines (20 to 80), green dashed lines (200 to 800), and red solid lines (2000 to 8000). Additional simulations were performed for smaller cluster sizes, and these results are shown in black.

If CNT were correct, all the data points shown in Figure 1 would fall onto a straight line with a slope of ${\left(\frac{36\pi }{\rho^{2}}\right)}^{1/3}\gamma$ and an intercept of 4 Δ*μ*. However, this was not observed for either the Lennard-Jones or the TIP4*P*/2005 water system. Figure 1 also shows the linear fits to the Δ^2^ *G* results across three distinct cluster size ranges, specifically 20 to 80, 200 to 800, and 2000 to 8000. For Lennard-Jones, the slopes (surface tension) obtained are 7.494 ± 0.002 (0.9680 ± 0.0002), 9.0931 ± 0.0005 (1.17457 ± 0.00006), and 9.077 ± 0.005 (1.17241 ± 0.00007) for the three cluster size ranges. For TIP4*P*/2005 water, the slopes (surface tension) obtained are 8.73 ± 0.01 (77.4 ± 0.1 mN/m), 8.29 ± 0.02 (73.5 ± 0.2 mN/m), and 8.00 ± 0.10 (71.0 ± 0.9 mN/m) for these three cluster size ranges. For Lennard-Jones, both the slope and intercept show signs of convergence toward large clusters (which agrees with previous studies on this system^55,56^ ), whereas for TIP4*P*/2005 water, no such convergence is observed. For the water system, the slope or surface tension decreases as the cluster size increases within the range considered.

To examine whether the size-dependence of the surface tension can be described by the Tolman equation, the CNT equation was modified to include a size-dependent surface tension term,

$$\Delta G\left(n\right)=n\Delta \mu +n^{2/3}{\left(\frac{36\pi }{\rho^{2}}\right)}^{1/3}\gamma^{\infty }\left(1-\frac{2\delta }{R}\right)=n\Delta \mu +n^{2/3}{\left(\frac{36\pi }{\rho^{2}}\right)}^{1/3}\gamma^{\infty }-n^{1/3}{\left(\frac{384\pi^{2}}{\rho }\right)}^{1/3}\gamma^{\infty }\delta$$ 4

which would yield the following Δ^2^ *G*

$$\Delta^{2}G=4\Delta \mu +\left[{\left(n+2\right)}^{2/3}-{\left(n-2\right)}^{2/3}\right]{\left(\frac{36\pi }{\rho^{2}}\right)}^{1/3}\gamma^{\infty }-\left[{\left(n+2\right)}^{1/3}-{\left(n-2\right)}^{1/3}\right]{\left(\frac{384\pi^{2}}{\rho }\right)}^{1/3}\gamma^{\infty }\delta$$ 5

Building on the Δ^2^ *G* analysis, we introduce a related constructed quantity, Δ^3^ *G*, defined as a difference between Δ^2^ *G* terms evaluated at two distinct sets of cluster sizes as follows

$$\Delta^{3}G=\left[\Delta G\left(n+2\right)-\Delta G\left(n-2\right)\right]-\left[\Delta G\left(n/10+2\right)-\Delta G\left(n/10-2\right)\right]=\{{[\left(n+2\right)}^{2/3}-{\left(n-2\right)}^{2/3}]-\left[{\left(n/10+2\right)}^{2/3}-{\left(n/10-2\right)}^{2/3}\right]\}{\left(\frac{36\pi }{\rho^{2}}\right)}^{1/3}\gamma^{\infty }-\left\{\left[{\left(n+2\right)}^{1/3}-{\left(n-2\right)}^{1/3}\right]-\left[{\left(n/10+2\right)}^{1/3}-{\left(n/10-2\right)}^{1/3}\right]\right\}{\left(\frac{384\pi^{2}}{\rho }\right)}^{1/3}\gamma^{\infty }\delta =a\left(n\right){\left(\frac{36\pi }{\rho^{2}}\right)}^{1/3}\gamma^{\infty }-b\left(n\right){\left(\frac{384\pi^{2}}{\rho }\right)}^{1/3}\gamma^{\infty }\delta$$ 6

This combination is designed to isolate the contributions of *γ* ^∞^ and *δ*. Based on the Tolman equation, a plot of Δ^3^ *G*/*a*(*n*) versus *b*(*n*)/*a*(*n*) is expected to fall on a straight line with an intercept of ${\left(\frac{36\pi }{\rho^{2}}\right)}^{1/3}\gamma^{\infty }$ and a slope of ${-\left(\frac{384\pi^{2}}{\rho }\right)}^{1/3}\gamma^{\infty }\delta$ . Estimating Δ^3^ *G*/*a*(*n*) requires highly precise Δ*G* and Δ^2^ *G* values, which can be difficult to achieve with limited simulation length. To mitigate noise and improve reliability, we therefore select cluster pairs that are well separated in size. Figure 2 shows how Δ^3^ *G*/*a*(*n*) varies with *b*(*n*)/*a*(*n*) for these two systems. For both LJ and water, this dependency demonstrates a clear linear behavior, particularly for larger clusters. For LJ, using a linear fit on the data points obtained for the three largest clusters, the intercept (*γ* ^∞^) and slope (*δ*) values are 9.072 ± 0.005 (1.1718 ± 0.0006) and 0.18 ± 0.03 (−0.0066 ± 0.0013), respectively. For water, using a linear fit for all clusters except the smallest one, the intercept (*γ* ^∞^) and slope (*δ*) values are 7.7 ± 0.1 (68.4 ± 0.9 mN/m) and 3.8 ± 0.6 (−0.48 ± 0.07 Å), respectively. For both systems, *γ* ^∞^ values, determined through other methods, are also shown for comparison. For LJ, a *γ* ^∞^ value of 1.1718 ± 0.0006 is extrapolated through this linear fit, which agrees well with a value of 1.18 ± 0.01 obtained from finite-size scaling techniques and grand-canonical transition-matrix Monte Carlo simulations for an infinite system.^57^ For TIP4*P*/2005 water, a *γ* ^∞^ value of 68.4 ± 0.9 mN/m is extrapolated from this linear fit, aligning closely with a value of 68.2 ± 0.3 mN/m obtained from the surface tension dependence on the van der Waals cutoff radius (*r*_vdW_) and several simulations at different *r*_vdW_ values.^58^

**Figure 2:**
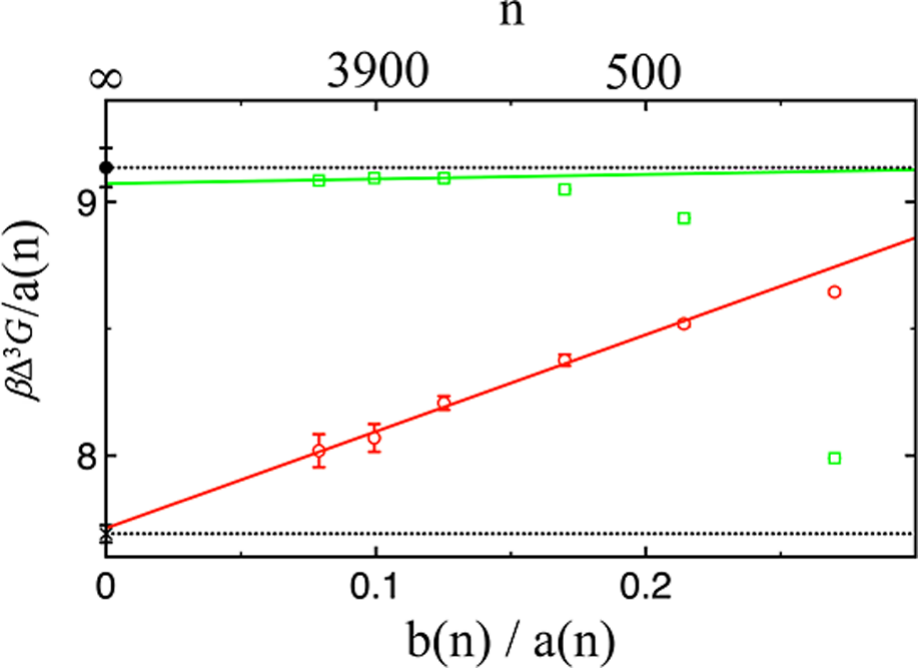
Δ^3^ *G*/*a*(*n*) in units of *k*_B_ *T* as a function of *b*(*n*)/*a*(*n*) obtained for Lennard-Jones (squares) and TIP4*P*/2005 water (circles). Linear fits are shown as solid lines, performed where the data begin to fall onto a straight line: for Lennard-Jones, this is over the three largest clusters, and for TIP4*P*/2005 water, it is for all clusters except the smallest one. According to eq 6, the intercept obtained from this linear fit corresponds to ${\left(\frac{36\pi }{\rho^{2}}\right)}^{1/3}\gamma^{\infty }$ . The dotted black lines and symbols along the *y*-axis indicate the location of *γ* ^∞^, as determined by bulk-phase simulation approaches for Lennard-Jones (filled circles)^49^ and TIP4*P*/2005 water (crosses),^50^ scaled by *a* factor of ${\left(\frac{36\pi }{\rho^{2}}\right)}^{1/3}$ .

For LJ, negative Tolman length (or δ values) were also found in c-DFT or mean-field studies^18–20,59–62^ whereas molecular dynamics simulations by Haye and Bruin^63^ yielded a positive value. For water, conflicting results have been reported, as discussed above. However, the value of −0.48 ± 0.07 Å extrapolated from this simulation study closely matches several of these studies. For instance, using the Young–Laplace relation, Leong and Wang^27^ obtained a value of −0.48 Å for a different water model at 298 K. Joswiak et al.^11^ found a value of −0.56 ± 0.9 Å for TIP4*P*/2005 at 300 K using the mitosis method. Using c-DFT, Wilhelmsen et al.^21^ estimated a value of −0.5 Å. Azouzi et al.^64^ reported −0.47 Å from cavitation experiments in quartz inclusions at ∼320 K. In contrast, using molecular dynamics simulations and the test-area method for pressure calculations on the TIP4*P*/2005 water model at 293 K, Lau et al.^25^ found that the surface tension for droplets displayed a sharp decrease from the planar limit, implying a positive Tolman length with no explicit value reported. The Tolman length was also shown to be positive for this model by Malek et al.^26^ from molecular dynamics simulations, in the range from 2 to 3 Å, when using both mechanical and thermodynamic approaches to pressure calculations.

Although direct measurements of surface tension or surface free energy for cluster systems remain challenging, experimental nucleation rates at 300 K suggest that, for water the droplet surface free energy is approximately 5 mJ/m^2^ higher than that of a planar surface.^11,65–67^ Also, the nucleation barrier height extrapolated from the experimental data at 300 K is about 5.9 *k*_B_ *T* higher than the one predicted by CNT.^65–67^ To compare with these experimental findings, additional simulations were performed to compute the nucleation free energy of clusters continuously up to a cluster size of 150 at a supersaturation of 3, which is expected to yield a nucleation rate comparable to the experimental measured rate range for this water model. It is shown in Figure 3 that the CNT underestimates the barrier height by about 5.6 *k*_B_ *T*.

**Figure 3:**
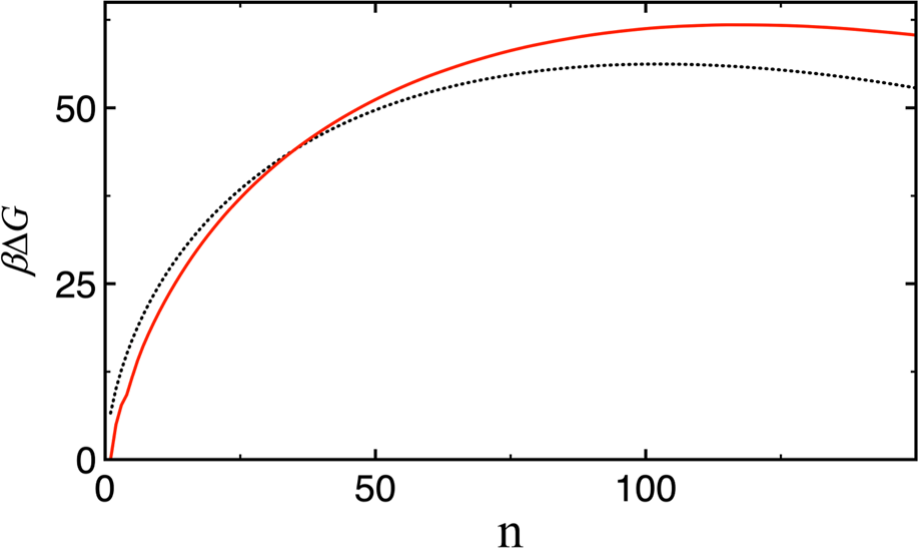
Δ*G* in units of *k*_B_ *T* as a function of *n* obtained for TIP4*P*/2005 water from the simulation (red solid line) and from CNT (black dotted line) using eq 1.

## 4 Conclusions

In summary, extensive aggregation-volume-bias Monte Carlo simulations were performed over an unprecedentedly large range of cluster sizes for both Lennard-Jones and TIP4*P*/2005 water to investigate the applicability of two fundamental theories in predicting cluster thermodynamics, i.e., classical nucleation theory and the Tolman equation. For both systems, the simulation results directly support that these two theories can be applied to clusters ranging from large sizes down to those containing just a few hundred particles. While the Lennard-Jones system exhibits a relatively weak size dependence of surface tension, the size-dependence effect is crucial for the water system, where the Tolman equation accurately describes this behavior for large clusters, yielding a negative Tolman length of −0.48 ± 0.07 Å. However, both theories break down for small clusters. The simulation results reported here have profound implications. First, small clusters containing a few hundred particles follow the bulk-droplet thermodynamic behavior already and the properties obtained from these small clusters can be used to interpolate or extrapolate how clusters of any sizes, including the infinite size or the bulk phase, would behave thermodynamically, via the use of CNT and the Tolman equation. Second, deviation from this bulk-droplet behavior occurs at the smallest clusters, which can be modeled using fully atomistic models. These findings lay the groundwork for a unified theoretical and computational framework capable of predicting cluster properties from monomers to the bulk phase.
